# Bridging Structure- and Ligand-Based Virtual Screening through Fragmented Interaction Fingerprint

**DOI:** 10.1021/acsomega.4c05433

**Published:** 2024-09-03

**Authors:** Rezi Riadhi Syahdi, Swarit Jasial, Itsuki Maeda, Tomoyuki Miyao

**Affiliations:** † Graduate School of Science and Technology, Nara Institute of Science and Technology, 8916-5 Takayama-cho, Ikoma, Nara 630-0192, Japan; ‡ Data Science Center, Nara Institute of Science and Technology, 8916-5 Takayama-cho, Ikoma, Nara 630-0192, Japan

## Abstract

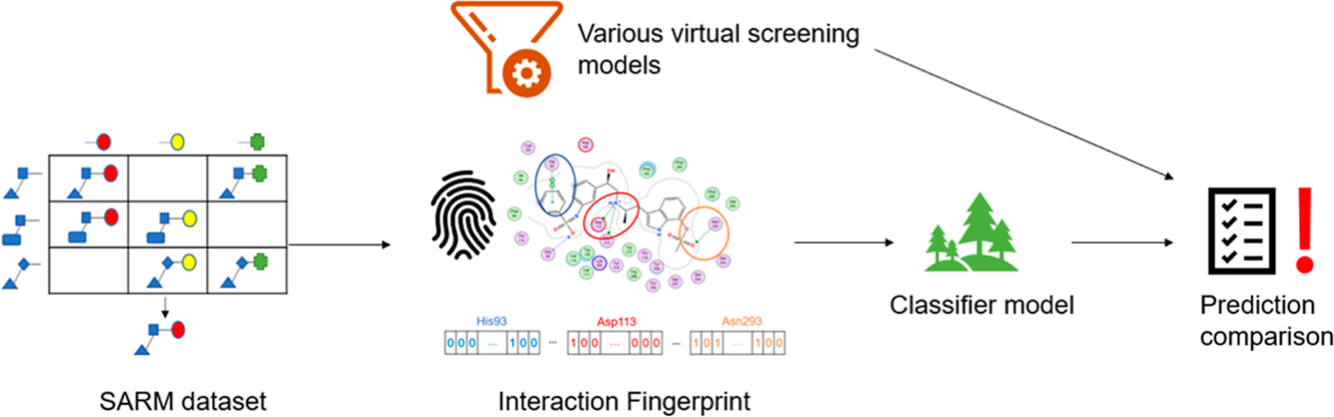

Ligand-based virtual screening (LBVS) and structure-based virtual screening (SBVS), and their combinations, are frequently conducted in modern drug discovery campaigns. As a form of combination, an amalgamation of methods from ligand- and structure-based information, termed hybrid VS approaches, has been extensively investigated such as using interaction fingerprints (IFPs) in combination with machine learning (ML) models. This approach has the potential to prioritize active compounds in terms of protein–ligand binding and ligand structural characteristics, which is assumed to be difficult using either one of the approaches. Herein, we present an IFP, named the fragmented interaction fingerprint (FIFI), for hybrid VS approaches. FIFI is constructed from the extended connectivity fingerprint atom environments of a ligand proximal to the protein residues in the binding site. Each unique ligand substructure within each amino acid residue is encoded as a bit in FIFI while retaining sequence order. From the retrospective evaluation of activity prediction using a limited number and variety of active compounds for six biological targets, FIFI consistently showed higher prediction accuracy than that using previously proposed IFPs. For the same data sets, the screening performance of LBVS, SBVS sequential VS, parallel VS, and other hybrid VS approaches was investigated. Compared to these approaches, FIFI in combination with ML showed overall stable and high prediction accuracy, except for one target: the kappa opioid receptor, where the extended connectivity fingerprint combined with ML models showed better performance than other approaches by wide margins.

## 1 Introduction

Virtual screening (VS) is a daily routine in modern drug discovery to predict bioactive compounds from large compound libraries.^1,2^ It is a common understanding that structure-based VS (SBVS) is preferable when the three-dimensional (3D) binding site of the target macromolecule is available, which usually employs molecular docking due to its computational efficiency. Nonetheless, ligand-based VS (LBVS) also shows promising results for practical applications.^3−6^ These two approaches can be naturally combined to efficiently identify bioactive compounds. For example, LBVS followed by SBVS is a standard to identify bioactive compounds.^7−10^ Recently, other forms of combinations: parallel VS and hybrid VS were also conceptualized.^11^ Parallel VS employs ligand- and structure-based approaches simultaneously and derives the final ranking of compounds.^12^ On the other hand, there is also the hybrid VS method, which merges ligand- and structure-based approaches at a methodological level. Traditionally, information about a bound ligand was willingly incorporated in molecular docking simulations in many forms to improve their scoring functions. For example, docking poses with high similarity to the bound conformation of a ligand can be prioritized.^13−16^ Similarly, Anighoro and Bajorath reported better SBVS performances when a 3D similarity to a reference ligand was considered.^17^ Although these methods significantly improve the docking simulation quality (reconstruction accuracy and screening performance), only bound ligand conformations are considered.

To use bioactive compounds with simulated conformation, i.e., docked poses, rescoring docking energy based on the output of a neural network model using both molecular structural and interaction information has been proposed, which can be regarded as a hybrid VS method.^18,19^ Although this approach automatically predicts bioactivity from a docked complex, a large number of active/inactive compounds are required to train the neural networks, preventing their applications for hit discovery projects where a small number of bioactive compounds are available.

Interaction fingerprints (IFPs) represent an interaction pattern between a ligand and a target macromolecule,^20^ forming a bit vector representing the presence/absence of a specific interaction with the macromolecule’s residues. Because IFPs are a natural representation of protein–ligand interaction, they can be utilized for binding mode prediction,^21^ rescoring of docking scores,^22^ and the prediction of binding affinity in combination with machine learning (ML) models.^23,24^ The classification and applications of IFPs were reviewed by Wang et al.^25^ Some variants of IFPs incorporate ligand-substructure information explicitly, thus they can be utilized for hybrid VS approaches.^26−28^ One of the successful IFPs is protein–ligand interaction profiler (PLIP) developed by Salentin and team.^29^ They identified new indication of amodiaquine, a malaria drug, as an anticancer agent using the IFP.^30^ The IFP of structural interaction fingerprint (SIFt), which incorporates seven interaction bits, was used as a postdocking molecular filter.^31^ de Graaf et al. incorporated this IFP in their drug discovery scheme to establish novel compounds with activity against human histamine H1 receptor.^32^ Another example is the protein atom contribution IFP, which uses weighting factors reflecting the relative frequency of specific interactions, improved results with better ligand positioning, and the early enrichment in combination with the GOLD scoring function CHEMPLP.^33^ One of the most recent IFPs is protein–ligand extended connectivity (PLEC), PLEC showed consistently superior results against comparison fingerprints.^28^ The PLEC fingerprint is constructed from hash values, each of which corresponds to a pair of substructures of the ligand and residue within a distance of 4.5 Å. These substructures were identified by expanding a pair of atoms involved in molecular interaction to the surrounding atomic environment with a predefined topological length on the chemical graphs. In their retrospective study, the prediction accuracy of binding affinity using the PDBbind v2016 data set^34^ was consistently higher than other IFPs, including structural protein–ligand IFPs.^27^ Extended IFP (EIFP) is another IFP, which is conceptually similar to extended connectivity fingerprints (ECFP), widely used ligand-based structural fingerprints.^35^ EIFP employs geometrical distance as a metric for the extension of substructures within a binding site instead of topological distance.^36^ Compared with several IFPs in combination with deep neural network models for docking score prediction, PLEC was slightly better than EIFP. Although PLEC can be regarded as state-of-the-art in IFPs, it does not represent the information on the order of amino acids within the fingerprint, which is important in particular when multiple but the same type of interactions appear.

Herein, we developed an IFP, termed the fragmented interaction fingerprint (FIFI), and a hybrid VS workflow using FIFI in combination with an ML model. FIFI takes ECFP as its basis and adds a layer of complexity to the ligand structure against amino acid residues from the binding sites. The major difference between FIFI and PLEC is that a hash number or a bit in FIFI is assigned on a residual basis, meaning that the same type of interaction but involved in different residues is recognized as different. As a retrospective validation for FIFI, we assumed to conduct of VS in the hit identification stage or the early stage of a hit-to-lead campaign, where a small number of structurally similar compounds are synthesized and tested. At this stage, identifying additional bioactive compounds, in particular, structurally distinct from current bioactive compounds, is important but challenging because of the limited information on active compounds. For that purpose, we utilized previously curated publicly available VS data sets^37^ focusing on six biological targets: beta-2 adrenergic receptor (ADRB2), caspase-1 (Casp1), kappa opioid receptor (KOR), lysosomal alpha-glucosidase (LAG), MAP kinase ERK2 (MAPK2), and tumor suppressor protein p53 for which we could access X-ray cocrystallized structures in the Protein Data Bank (PDB) database^38^ and the data sets were organized as clustered based on structurally similar compounds. Using these data sets, we evaluated prediction performances of various VS approaches, focusing on LBVS, SBVS, and their combination approaches, including FIFI-based approaches.

## 2 Materials and Methods

### 2.1 VS Data Sets

Compound data sets were compiled from previously published data for benchmarking VS performance using various ligand-based approaches.^37^ These data sets consisted of bioactive compounds extracted from the ChEMBL database and inactive compounds from the PubChem database.^39,40^ Training active compounds were organized in the form of structure–activity relationship matrices (SARMs),^41^ where compounds on each row form a matching molecular series and the cores of rows have matched molecular pair relations.^42,43^ Test active and inactive compounds were diverse compounds extracted from the databases. From the 15 target macromolecules in the original data, six diverse macromolecular targets were selected in this study, for which X-ray cocrystallized structures were available. These targets represent a distinctive prominent set of drug screening targets such as the GPCR, kinases, protease, hydrolase, and transcription factor families.^44−46^

SARMs contained 10–50 active compounds, and representative SARMs were selected based on hierarchical clustering to reduce the bias of selecting similar SARMs as training as explained by Maeda et al.^37^ Furthermore, the SARMs containing more than 10 active compounds that had specified stereochemistry were selected as training data sets to avoid the stereo ambiguity of compounds. The overall profiles of target-wise data sets are reported in Table 1 and that of each SARM in Supporting Information Table S1. For each SARM, two test data sets were prepared: whole and distinct test data sets. Whole test data sets consisted of active and inactive compounds not residing in the training SARMs, while distinct test data sets contained compounds with a similarity value of less than 0.2 to the nearest training compounds (the number of compounds is given in Supporting Information Table S2). The similarity metric was the Tanimoto coefficient using ECFP4 2048 bits. The number of test compounds depending on the similarity threshold is shown in Table 2.

**Table 1:** Data Set Profiles^a^

target	abbreviation	UniProtKB ID	# active CPDs	# inactive CPDs	# SARMs	# average active CPDs per SARM
beta-2 adrenergic receptor	ADRB2	P07550	761	114,669	6	30
caspase-1	Casp1	P29466	1682	32,856	6	25
kappa opioid receptor	KOR	P41145	1929	117,824	14	22
lysosomal alpha-glucosidase	LAG	P10253	6358	163,088	11	14
MAP kinase ERK2	MAPK2	P28482	1792	38,781	6	24
cellular tumor antigen p53	p53	P04637	7738	119,738	10	24

a For each target, data set profile is reported including the abbreviation, UniProtKB ID for the target. The numbers of active compounds (CPDs), inactive CPDs, SARMs, and the average active CPDs per SARM are reported.

**Table 2:** Test Active and Inactive Compounds per Similarity to the Training Data Sets^a^

	# active CPDs				# inactive CPDs			
threshold	0.15	0.20	0.30	-	0.15	0.20	0.30	-
ADRB2	40	184	530	731	41,224	86,416	103,845	104,669
Casp1	352	959	1328	1657	8537	20,064	25,042	25,406
KOR	583	1328	1781	1907	42,735	84,948	101,619	102,224
LAG	3084	5259	6188	6345	70,057	127,825	151,227	153,119
MAPK2	78	502	1288	1768	11,817	26,896	31,414	31,481
p53	1163	4030	7157	7714	19,314	53,776	85,468	90,000

a The average number of active and inactive compounds (CPDs) in the test data sets is reported using various threshold values of Tanimoto similarity to the training data sets. The similarity thresholds are varied from 0.15 to 0.30, or without introducing any similarity constraints (-).

### 2.2 Fragmented Interaction Fingerprints

**Figure 1:**
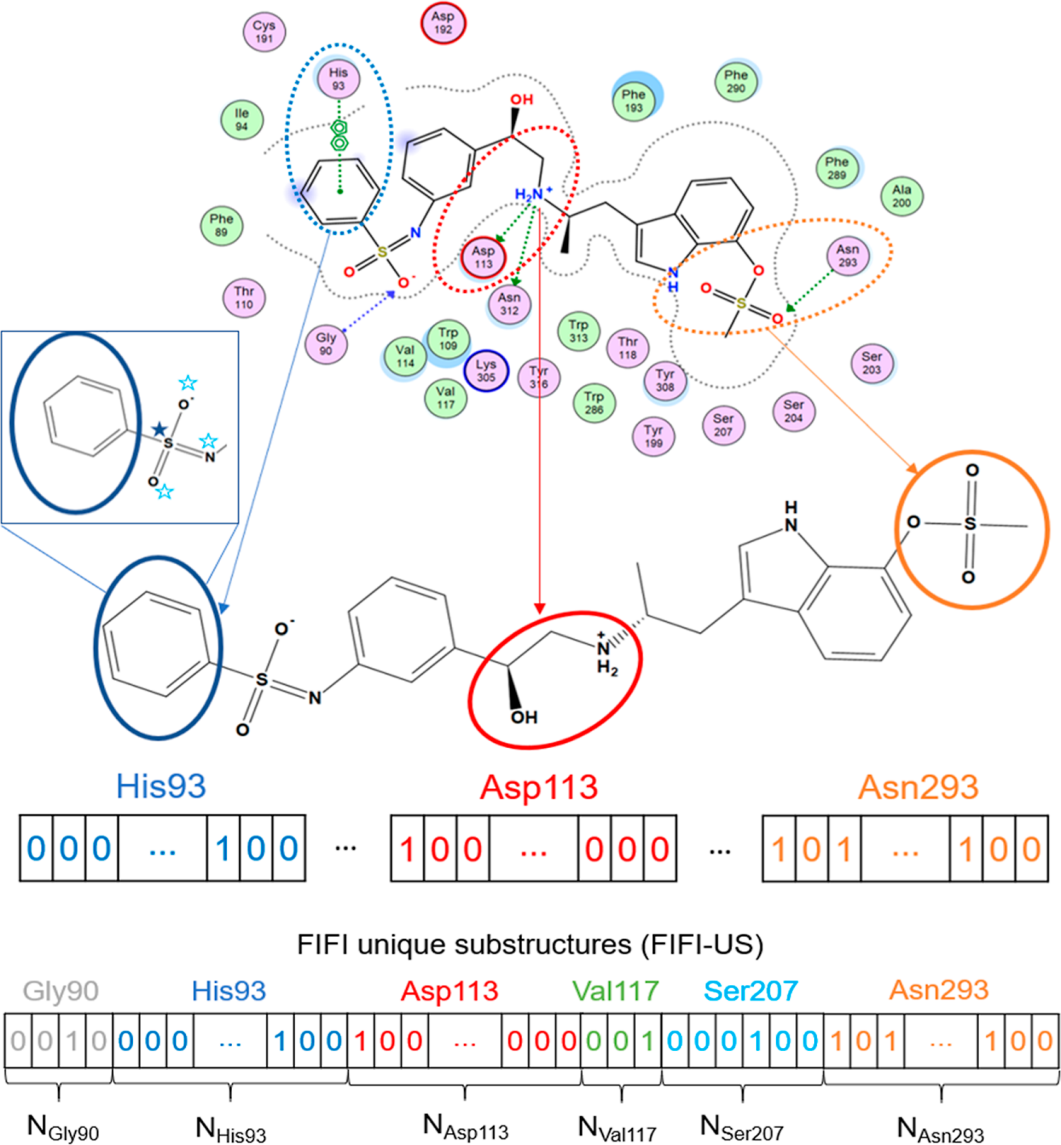
FIFI generation scheme. The 3D structure of a ligand from the docking pose was used as the input. For each residue in the binding site, the ligand’s atoms and bonds within the vicinity were extracted (circles). They can be extended to the atom neighbors (star: 1 bond neighbor, hollow star: 2 bond neighbor in the blue square). These atoms and bonds (substructures) are translated into bits in fingerprints, ordered by the amino acid residue. The substructures were ECFP patterns, and the translation forms a concatenated ECFP2 bit array (FIFI-BA) or unique substructures (FIFI-US). N is the number of unique bits based on the ligands’ substructure within each amino acid vicinity.

FIFI is a ligand-substructure incorporated IFP like PLEC.^28^ The major difference between FIFI and PLEC is that FIFI has information about the order of amino acid residues in its fingerprint form, while PLEC does not. This feature makes FIFI reasonable, in particular, to distinguish the same interaction pair yet different residual numbers. FIFI is generated from a docked ligand structure, currently from the SDF file format. The generation scheme of FIFI is illustrated in Figure 1. For each amino acid in the binding site, proximal atoms in the ligand structure are identified as tagged atoms. The distance threshold of proximity was defined as 5.5 Å for incorporating important contacts (i.e., hydrogen bonds 2.2–4.0 Å and salt bridge <4.0 Å).^47^ Many models define interatomic contacts using a distance threshold of 4.5 Å,^48,49^ and 1 Å additional distance margin was added for compensating potential nonoptimized docking poses. The tagged atom list can also be extended by adding neighboring atoms in the ligand structure. In this study, we compared no neighbor (N_0_), 1 bond neighbor (N_1_), and 2 bond neighbor (N_2_) as neighbor inclusion configurations. These tagged atoms were subsequently translated into fingerprints in two forms: FIFI bit array (FIFI-BA) and FIFI unique substructures (FIFI-US). In both forms, tagged atoms are first translated to ECFP hash values corresponding to unique substructures. FIFI-BA consists of a concatenation of fixed-sized ECFP bit vectors for amino acid residues while preserving the order of amino acid residues. In this representation, hash collision (multiple hash values are mapped to the same bit in an array) may occur. However, FIFI-US was made through a one-hot-encoding translation for hash values. This results in one-hot-encoded fingerprints on an amino acid residual basis that are concatenated in the order of amino acid residues. FIFI-US can avoid bit collision but lose the information of nonexisting substructures. For FIFI-BA, 1024 bits per amino acid residue were used in this study. The VS workflow using FIFI can be regarded as a hybrid VS approach in this study.

### 2.3 VS Approaches for Comparison

#### 2.3.1 Evaluation Procedure

**Figure 2:**
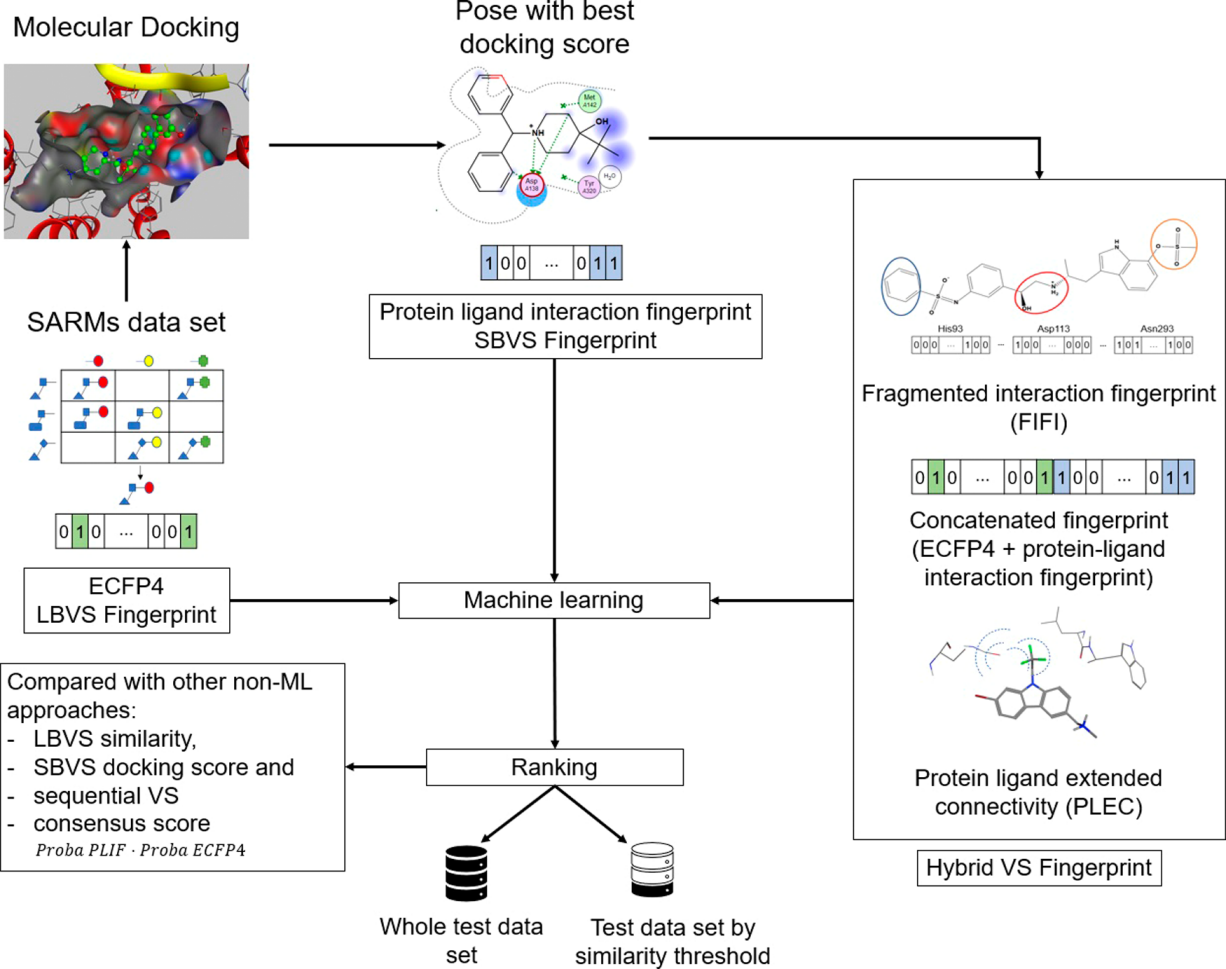
VS evaluation workflow. From each SARM data set, we compared various approaches using ML or without using ML. Various fingerprints were used as input for the ML approach: ECFP4 as LBVS fingerprint, PLIF as SBVS fingerprint, concatenated ECFP4-PLIF, PLEC and FIFI as hybrid VS fingerprint.

For a fair evaluation of VS performance using FIFI in combination with ML models, various VS approaches were compared. The evaluation scheme is shown in Figure 2. Four non-ML methods and five ML methods were tested. As non-ML approaches, similarity searching based on Tanimoto similarity was employed for LBVS and docking score-based ranking for SBVS. The molecular representation for ligand-based approaches was ECFP4 in the form of a 2048-dimensional bit vector. As a sequential approach of LBVS and SBVS, Tanimoto similarity-based similarity searching, followed by ranking based on the docking score, was tested. As a parallel approach, a consensus score from protein-ligand IFP(PLIF) and ECFP4 was also tested, which is a simple multiplication between the two scores. When ML models were employed, ECFP4 was used for LBVS, PLIF for SBVS, and the concatenation of these two representations was used as a hybrid approach. Focusing on IFPs, we compared FIFI to a previously published fragmentation fingerprint technique: PLEC, which showed high VS performance in previous studies.^28,36^

#### 2.3.2 Ligand Conformer Generation

In this study, ligand conformers were generated using in-house Python script utilizing OpenEye OMEGA tools resulting in a 3D structure with *.sdf file format.^50^ Only compounds with unique configurations were considered from the original data set.^37^ An energy-minimized conformer was generated for each molecule in the curated database according to the MMFF94s force field.^51^ The parameters were as follows: the root-mean-square Cartesian distance of atomic position (RMSD) threshold was set to 1.0, and a strict definition of atom typing was used, where a molecule is rejected if any atom has an invalid atom type. The molecule charges and protonation state were then determined using the Protonate 3D module in Molecular Operating Environment (MOE) software.^42^

#### 2.3.3 Molecular Docking Score

Molecular docking was conducted using MOE software.^42^ Macromolecule structures were retrieved from the PDB for each target.^38^ They were selected based on the bounded ligand, resolution, and *R*-value. The preparation steps included removing the solvent moieties farther than 4.5 Å from the binding sites, repairing structural errors through the QuickPrep module, adding hydrogen atoms, and optimizing the hydrogen position and charge through the Protonate 3D and Partial Charges modules in the MOE. The protonation states of ligands were then assigned by the Wash module in the MOE database.

Three variations of scoring refinement methods were tested: (1) without refinement, (2) rigid receptor refinement with London dG scoring, and (3) rigid receptor refinement with generalized Born volume integral (GBVI)/solvent accessibility (SA) dG. Docking scores were sorted, and molecules with lower docking scores were ranked as high. Two docking scores compared have different scoring approaches. The London dG scoring method is empirical, relying on experimental data to estimate binding free energies. In contrast, the GBVI/SA scoring method is based on a force-field approach, incorporating the GBVI and SA calculations.^42,52^ This comparison allows us to select better scores from these different approaches.

#### 2.3.4 Interaction Fingerprints

##### 2.3.4.1 Protein–Ligand Interaction Fingerprint^42^

PLIF is a fingerprint implemented in the MOE that summarizes the interactions between ligands and proteins. Interactions such as hydrogen bonding and ionic interactions are classified according to the residue of origin. A residue may participate in two categories of interactions: potential (energy-based) and surface (patch) contacts. We categorized the extracted PLIF as PLIF-A (using only potential contact information) and PLIF-B (using both potential contact and surface patch information). We also used the counts of specific-type interactions for the construction of two more PLIF types: PLIF-C (count of potential contacts) and PLIF-D (count of potential and surface contacts). However, for constructing concatenated IFP and consensus scoring, and for the comparison with other VS, we only used PLIF-B. The interactions extracted using PLIF are given in Table S3, and the extraction scheme is provided in Figure S1.

##### 2.3.4.2 Protein–Ligand Extended Connectivity

PLEC is a ligand-substructure incorporated IFP. Each pair of the interacted substructures between a ligand and an amino acid residue forms a hash value, like ECFP. Depths from interaction points for ligands and proteins are parameters in PLEC to define the degree to which specific substructures were considered in a fingerprint. PLEC was generated with the configuration of ligand depth set to 2 and protein depth set to 4. The bit number was set at 7653 as suggested by the original research. The distance threshold was set at 4.5 Å.^28^

#### 2.3.5 Sequential Approach

The sequential VS approach used in this study first filters screening compounds with an LBVS approach. Screening compounds are sorted by Tanimoto similarity against the nearest neighbor active compound in the training data set. In the LBVS approach, the top 1% of screening compounds was selected. These compounds were then sorted by their docking score, as in the SBVS.

#### 2.3.6 Parallel Approach: Consensus Score

The consensus score from LBVS and SBVS scores was derived by performing dot multiplication of the probability scores obtained from the ML results of ECFP4 and IFP as representatives of ligand- and structure-based approaches, respectively.

#### 2.3.7 Singular and Concatenated Fingerprints for ML

ML models were employed for ligand- and structure-based and combined fingerprints. For ligand-based fingerprints, ECFP4 2048 bits were used. In this study, PLIFs were constructed based on one-hot encoding of interaction types for each residue used, including the surface contact information as additional bits. The concatenated FP was created by simply concatenating ECFP4 and PLIF. As ML methods, logistic regression,^53^ support vector machine (SVM),^54^ and random forest (RF)^55,56^ were employed. In SVM, linear, radial-based functions, and Tanimoto similarity-based kernels were tested. The output of the prediction model is the probability of the reaction being active.

### 2.4 Model Performance and Interpretation

We used the area under the precision-recall curve (AUPRC) to summarize the model’s overall performance. A precision-recall curve is a plot of precision (number of correct positive predictions made) as the *y* axis and recall (number of correct positive predictions out of all positive predictions that are possible to make). In an imbalanced data set, like the SARM data set, the metric is focused on the positive class which is the minority class and unaffected by the majority class.^57^

For the FIFI model feature importance analysis, we employed Shapley additive explanations (SHAP). SHAP is based on the Shapley interaction index from a theory of player performance in a coalition game theory.^58^ The Shapley value for a feature is the average of the marginal contribution of the feature value from all possible combinations of features. Since RF was used which is a decision tree-based model, the tree SHAP algorithm was employed to generate SHAP values.^59^ The feature contributions from this SHAP value could then be mapped back for each atom and bond using a modified approach from previous research.^60^

### 2.5 Docking Score ML Data Sets

To evaluate FIFI as IFPs using comprehensive data sets, we also evaluated ML performance using PLIF, PLEC, and FIFI on previously published docking score ML data sets that include curated compounds with docked poses.^61^ From the 155 targets in the data sets, we selected 33 targets that contain over 1000 compounds each. Duplicate entries and incomplete data (e.g., missing SMILES or IC50 values) were removed. The compounds were then classified into 25 bins based on the IC50 distribution using quantile binning with lower bins corresponding to lower IC50 (hence higher activity). Bins 1–7 were designated as actives, while bins 11–25 were annotated as inactive. To eliminate possible ambiguity from marginal compounds (which may be classified as active or inactive), bins 8–10 were removed. RF was employed as a ML method, and 80–20% stratified split was conducted to form training and test data sets. Hyperparameters were optimized using 5-fold cross-validation using only the training data set. Detailed number of compounds and the IC50 criteria are provided in Supporting Information Table S4.

## 3 Results and Discussion

### 3.1 Docking Study

Molecular docking was conducted for all training and test compounds in this study to evaluate various ligand- and structure-based approaches. For selecting a trustful docking procedure, three refinement algorithms were tested based on redocking and VS performance, and the results are shown in Table 3. Most of the rmsd values between cocrystallized ligands and redocked ones were less than 2, which is generally considered an acceptable precision in docking.^49^ SBVS was conducted for the whole test data set to compare the ranking power of the rescoring method through the AUPRC, which serves as a proper parameter evaluation for highly imbalanced data sets. For ADRB2, Casp1, LAG and KOR, the London dG rescoring was the best, while the GBVI/SA rescoring was preferable for MAPK2 and p43. These preferable rescoring algorithms were used in the following study, where structure-based approaches were involved. Docking results are provided in Supporting Information in Figure S2 and Table S6.

**Table 3:** Redocking and Energy-Based VS^a^

		RMSD↓			AUPRC↑		
target	PDBID	NR	LDG	GBVI/SA	NR	LDG	GBVI/SA
ADRB2	6PS2	1.807	**0.608**	**0.608**	0.104	**0.295**	0.109
Casp1	1RWX	3.673	**1.217**	1.219	0.047	**0.057**	0.053
KOR	4DJH	1.438	1.458	**1.416**	0.053	**0.072**	0.023
LAG	5NN5	0.919	**0.579**	**0.579**	**0.046**	0.043	0.031
MAPK2	6G9N	2.747	2.207	**0.777**	0.044	0.040	**0.074**
p53	6SI3	1.282	**0.205**	0.206	0.070	0.073	**0.123**

a The RMSD between the bound (X-ray crystallized) and docked conformations of the template ligand specified by PDBID is reported for each target macromolecule. The VS performances for the whole test data set were measured based on the AUPRC. Three docking refinement algorithms in MOE were tested, NR: without refinement, LDG: refinement with the London dG rescoring, and GBVI/SA: with the GBVI/SA rescoring.

### 3.2 VS Performances

RF showed overall high prediction performance among the five tested ML algorithms when ECFP4 or PLIF was used as model input (Table S7). Thus, in the subsequent section, performances using RF will be reported when ML models are involved in the screening approaches.

#### 3.2.1 VS Performance for the Whole Test Data Sets

The VS performance in terms of AUPRC is summarized in Table 4. Overall, FIFI-US and FIFI-BA showed stable performance, as supported by the fact that FIFI-US was placed within the top 3 methods for all six targets. There was little difference in performance between FIFI-US and FIFI-BA. These FPs were superior to PLEC and the concatenated FPs. For the kappa opioid receptor, ECFP4 and the consensus score outperformed other approaches by a great margin. For this target, ligand information in combination with an ML model seemed crucial to prioritize active compounds against many inactive ones, supported by the fact that LBVS using similarity searching showed poor performance (0.092 in AUPRC). The necessity of ML models instead of similarity searching was consistent with our previous finding when identifying bioactive compounds from training compounds with limited diversity.^62^

**Table 4:** VS Performances for the Whole Test Data set^a^

	non-ML approaches				ML approaches					
target	LBVS (similarity)	SBVS (docking Score)	sequential (LBVS + SBVS)	parallel VS (consensus score)	ligand-based FP (ECFP4)	structure-based FP (PLIF)	hybrid VS FP			
							concatenated FPs	PLEC	FIFI-US	FIFI-BA
ADRB2	0.259	0.299	0.374	0.781	0.855	0.136	0.888	0.766	0.881	**0.891**
Casp1	0.233	0.061	0.322	0.238	0.308	0.080	0.299	0.281	0.322	**0.323**
KOR	0.092	0.237	0.118	0.684	**0.702**	0.163	0.248	0.332	0.362	0.313
LAG	0.042	0.045	0.052	0.051	0.054	0.041	0.049	0.054	**0.056**	0.052
MAPK2	0.576	0.094	0.576	0.609	0.701	0.115	0.747	0.555	**0.780**	0.767
p53	0.098	**0.134**	0.114	0.101	0.104	0.084	0.106	0.114	0.114	0.114

a Performances were measured in terms of the median value of the AUPRC. The highest value for each target is reported in bold.

Compared with different VS approaches, SBVS using docking score showed an overall poor performance. However, structure-based FP (PLIF) in combination with the ML model performed the worst. The performance of the parallel VS approach was between ligand- and structure-based FP approaches. Furthermore, different FIFI variants were found to have a comparative performance. However, due to the efficiency and the explainability of the bits, FIFI-US seemed more appealing to be used. On the other hand, it is noticeable from the result that the longer the included bond depth, the better the overall performance was. This may be explained by the fact that introducing a longer extension from the center atom lowers the uncertainty introduced by the pose generated by molecular docking. The comparison of neighbor extension for FIFI performance is given in Table S8.

#### 3.2.2 VS Performance for the Distinct Test Data Sets

VS performance for the distinct test data sets is summarized in Table 5. The distinct test data sets consisted of molecules with a maximum score of Tanimoto similarity below a threshold value of 0.2. Thus, structure-based approaches were expected to outperform their ligand-based counterparts. As expected, all approaches demonstrated lower performances for distinct test data sets. This result was aligned with the expected trend for ligand-based methods, but it was somewhat unexpected to observe a similar trend in structure-based approaches, especially SBVS using molecular docking scores. The docking scoring was performed irrespective of any training compounds; thus, uniform behavior throughout the test data set was expected. However, the possibility that these dissimilar active compounds exhibit different binding interactions or even binding sites could not be eliminated.

**Table 5:** VS Performances for the Distinct Test Data Sets (Similarity < 0.2)^a^

	Non-ML approaches				ML approaches					
							hybrid VS FP			
target	LBVS (similarity)	SBVS (docking score)	sequential (LBVS + SBVS)	parallel VS (consensus score)	ligand-based FP (ECFP4)	structure-based FP (PLIF)	concatenated FPs	PLEC	FIFI-US	FIFI-BA
ADRB2	0.029	0.055	0.019	0.330	0.347	0.025	0.321	0.187	0.349	**0.407**
Casp1	0.062	0.046	0.051	0.072	0.082	0.053	0.069	0.083	0.092	**0.097**
KOR	0.034	0.249	0.023	0.666	**0.714**	0.129	0.132	0.242	0.270	0.238
LAG	0.046	0.046	0.037	0.047	0.048	0.041	0.048	0.049	**0.055**	0.052
MAPK2	0.060	0.028	0.030	**0.288**	0.109	0.121	0.121	0.092	0.196	0.149
p53	0.100	**0.115**	0.078	0.079	0.082	0.070	0.082	0.091	0.079	0.080

a Performances were measured in terms of the median value of the AUPRC. The highest value for each target is reported in bold.

FIFI-US consistently showcased superior performance compared to the previously published fragmentation fingerprint: PLEC for the distinct test data sets except for p53. For this target, ML approaches did not demonstrate superior performance, as shown by the similarity of LBVS (0.100) versus ligand-based FP ECFP4 (0.082) or docking score SBVS (0.115) versus structure-based FP PLIF (0.070). In other targets, the superiority of FIFI compared to PLEC could be attributed to several differences, but the main factor could be the omission of amino acid residue order in bits folding. In PLEC, while each amino acid type was represented by different bits, the same type of residue with different positions was not explicitly differentiated. This is particularly critical for binding sites that are abundant in identical amino acid residues, for example, several phenylalanine and serine residues in the binding site of beta-2-adrenergic receptor.^63^

### 3.3 High VS Performance of Ligand-Based ML Models for KOR

**Figure 3:**
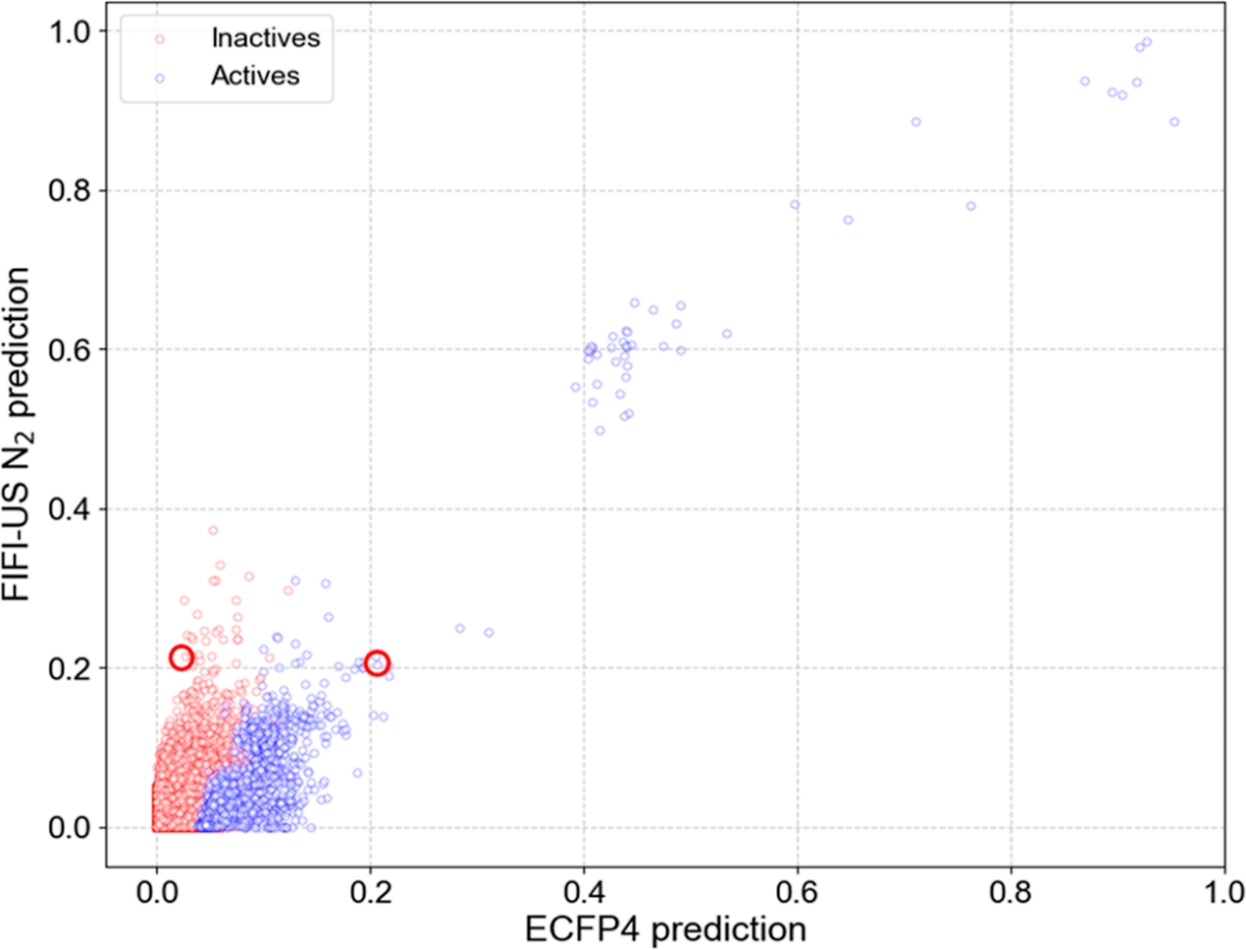
Prediction comparison of ECFP4 and FIFI-US N_2_ in KOR using 9595_series_1 SARM as training data. The *x* axis is the ECFP4-based prediction score (probability of being active), while the *y* axis is the FIFI-based prediction score. False positive prediction caused the lower FIFI performance in comparison to ECFP4. The binding modes of the circled two compounds are shown in Figure 4.

For both whole and distinct test data sets of KOR, RF models using ECFP4 as a descriptor showed a high VS performance by a wide margin. To understand possible reasons for lower performance by FIFI and all other VS with SBDD components, we compared ECFP4 and FIFI prediction head-to-head as illustrated in Figure 3. Low performance was primarily attributed to false positives, i.e., inactive compounds ranked higher than active ones. It was highly plausible that during the introduction of structure-based information, inactive compounds displayed confounding characteristics similar to those of active ones, compared to relying solely on ligand structural information. Removing false positives from molecular docking results has been one of the most challenging works in SBVS. This plaguing accuracy problem of the scoring function has been pointed out as a challenging issue many times.^64−66^

**Figure 4:**
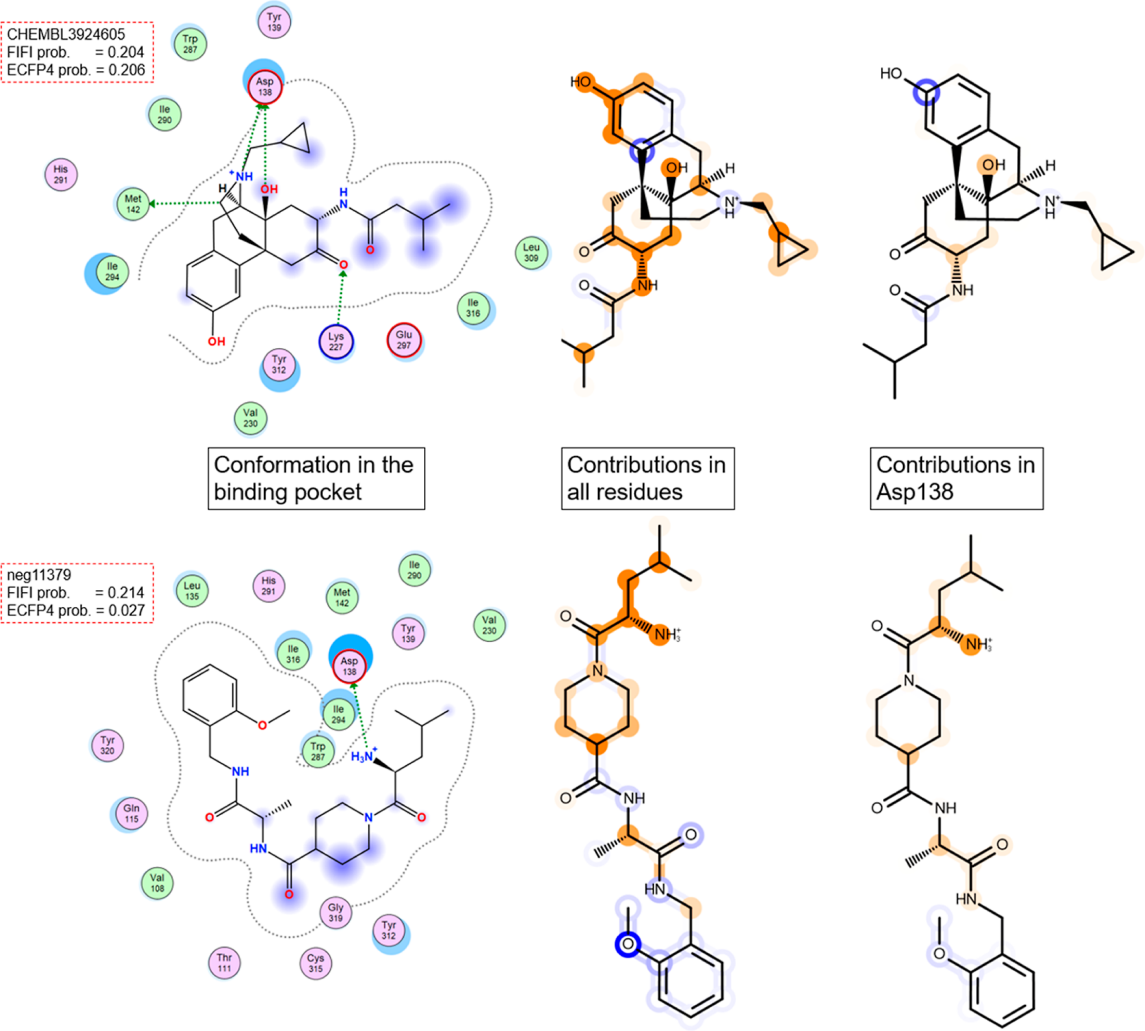
Exemplary pair of active (CHEMBL3924605) and inactive (neg11379) in KOR with similar FIFI probabilities and dissimilar ECFP4 probabilities is shown in red circles in Figure 3. The cumulative Shapley value for each atom is indicated by color (orange: positive contribution and blue: negative contribution). The cumulative contribution values are derived from all amino acid residues (center figure) or from only Asp138 contributions (right-hand figure).

A pair of active and inactive compounds having similar FIFI-based and dissimilar ECFP4 prediction values is reported in Figure 4. Since ECFP4 performed better than FIFI for the distinct test data set of KOR, substructural features of ligand compounds outweighed assumed interaction. To explain the FIFI model result, SHAP was employed and the cumulative contribution for each atom of the pair to the prediction score is visualized in Figure 4. We visualized the cumulative contributions of ligand substructure for all amino acid residues and only for Asp138, which has a critical role in the selectivity toward protonated amine-containing ligand.^67^ The positive contribution of the primary amine was observed in the negative control. This is in contrast to the negative contribution associated with the tertiary amine in the active control, even though both were shown to interact with Asp138. The training SARM contained active analogues with a primary amine substructure and not with a tertiary amine. In the case of the active compound, the hydroxyl part that interacted with Asp138 was highlighted as a positive contributor. However, the disparity in probability suggests that this substructure contribution was overshadowed by other parts’ contributions.

### 3.4 Characteristics of Top-Ranked Compounds

**Figure 5:**
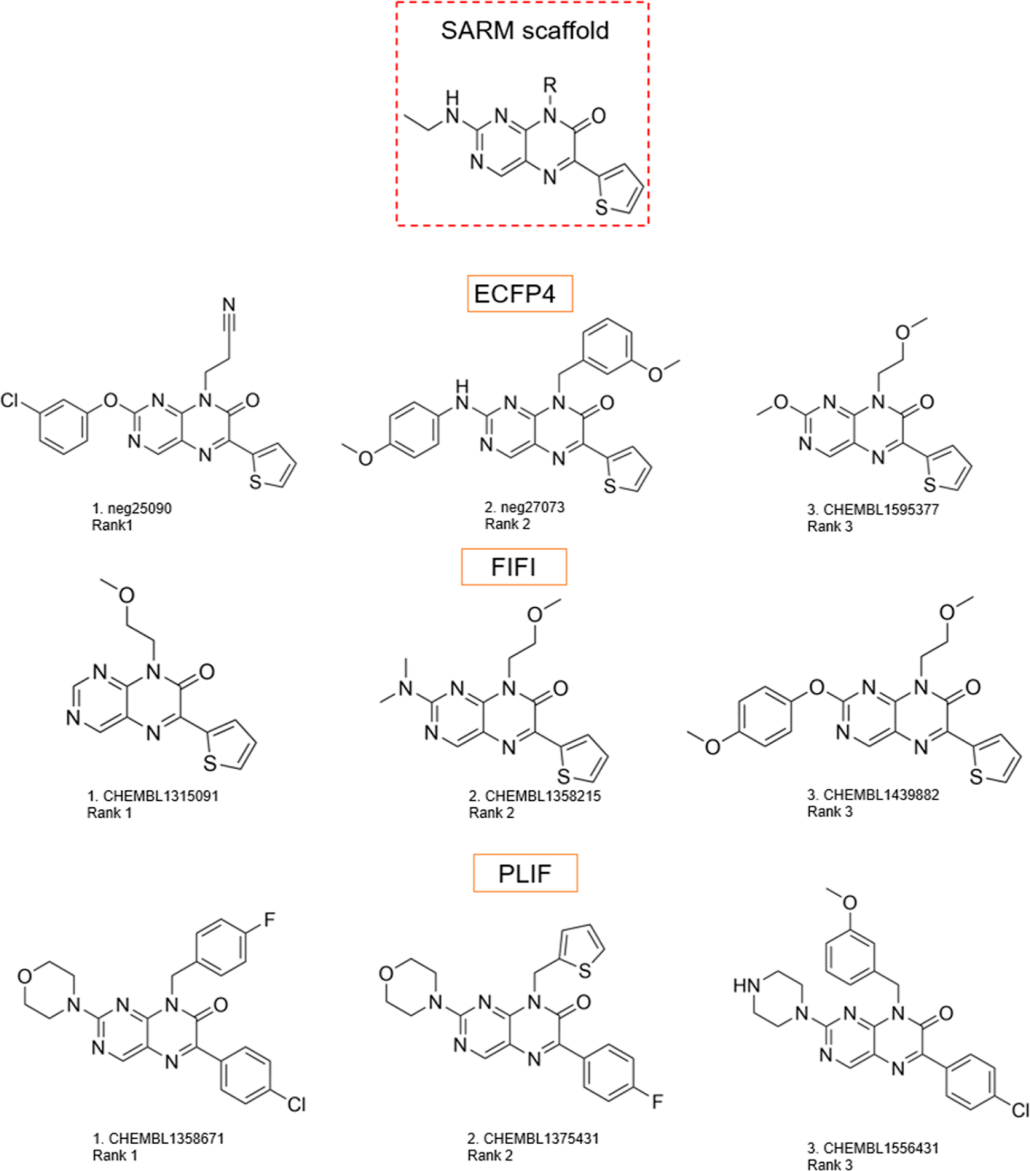
Top-ranked compounds of the whole test data set for Casp1. For example, the top three compounds for Casp1 are provided using SARM 5_series_1 as training data. For each compound and method, the ranking of compounds is also shown.

**Figure 6:**
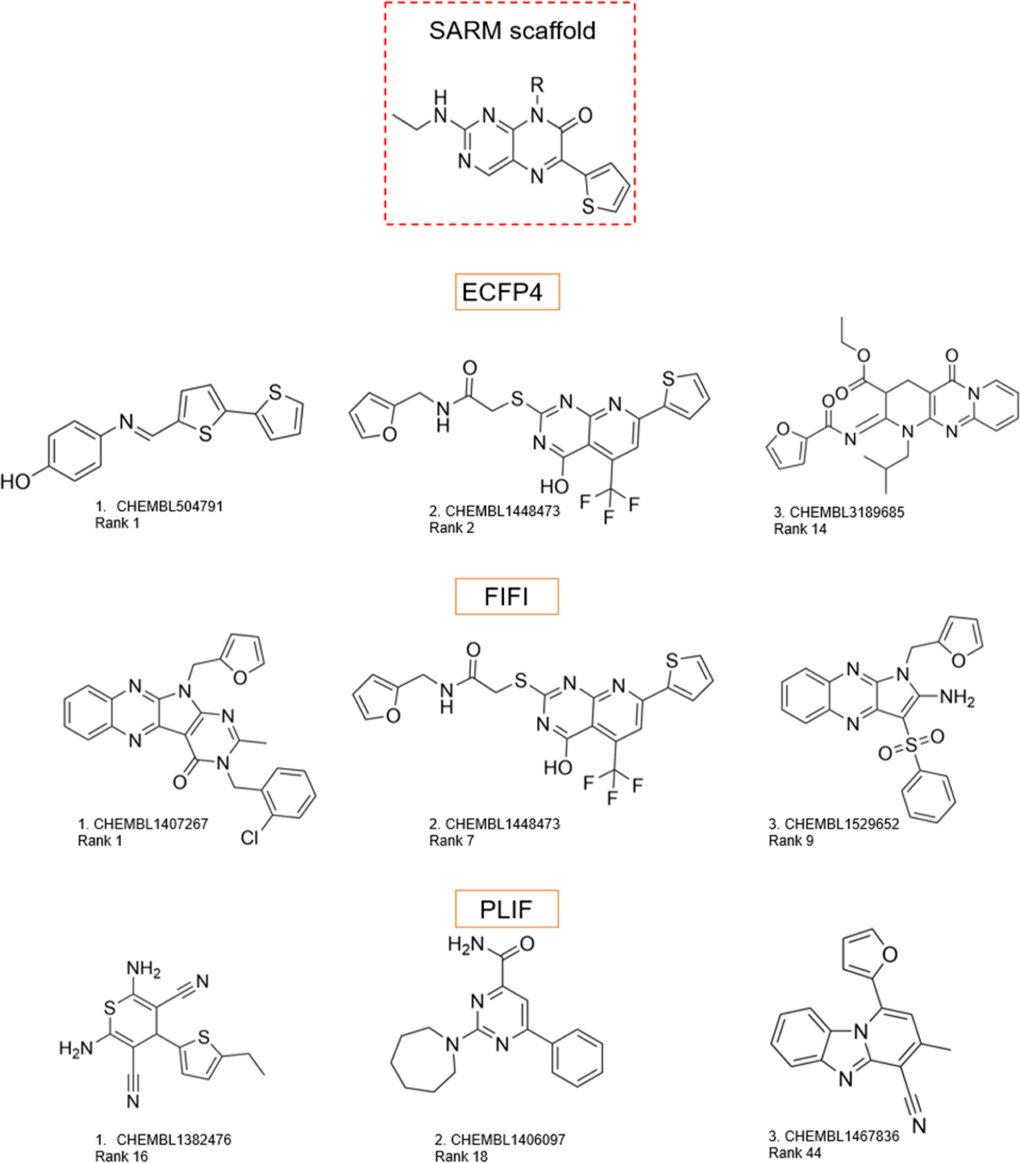
Top-ranked active compounds from the distinct test data set for Casp1. Top three active compounds from the distinct test data set are shown for Casp1 5_series_1 SARM. The rank for each compound is also reported below CHEMBL ID.

Top-ranked compounds were manually selected as representative cases to understand the differences in screened compounds among the methods. Figures 5 and 6 report the three top-ranked compounds for the whole test data set and the top-ranked active compounds for the distinct test data set, respectively. In these figures, a SARM of Casp1 was focused on as an example.

From the whole test data set as reported in Figure 5, all three ML models (PLIF, ECFP4, and FIFI) identified structures with a scaffold similar to that of training active compounds as the top-ranked compounds. These compounds were substituted pteridinone analogues, in the case of FIFI and ECFP4, the substituent was thiophene while for PLIF was halobenzene. Interestingly, the ECFP4- and PLIF-based ML model proposed compounds with different substituents in both N8 and C2, PLIF less varied in C2 with either a morpholine or piperazine moiety. Meanwhile, the top three compounds by the FIFI-based model only differentiated in C2 while retaining methoxyethyl substituent in N8. The first- and second-rank compounds by ECFP were tagged as inactive while all the top-ranked compounds by FIFI and PLIF were active.

To analyze the structural diversity of highly ranked compounds among the three methods, the number of unique Bemis–Murcko scaffolds was counted for the active compounds in the top 100 compounds. As reported in Figure S5, all three fingerprints—ECFP4, FIFI, and PLEC—exhibit similar performance in retrieving active scaffolds, with variations depending on specific targets. PLIF, however, consistently underperforms due to its lower accuracy in retrieving the actives.

For the distinct test data set, the PLIF- and ECFP4-based models proposed diverse compounds for its top three active compounds, as shown in Figure 6. Notably, the model using FIFI prioritized pyrroloquinoxaline analogues in two of the top three active compounds. Each FIFI-US bit encoded a unique substructure, enabling it to identify certain substructures in a highly probable position during binding with a proper target. In other words, these three analogues with reasonably similar substructures showed similarity in the docked pose and thus similar in size.

### 3.5 IFP Evaluation Using Docking Score ML Data Sets

**Figure 7:**
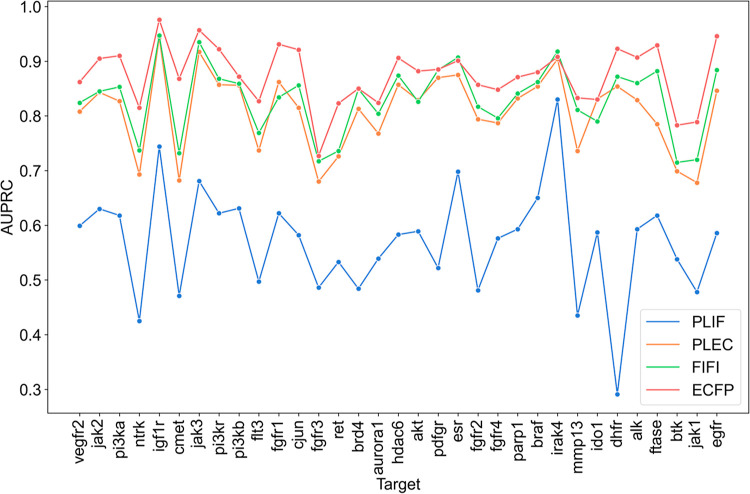
AUPRC for selected 33 targets from docking score ML data set.

A further evaluation of the screening ability of FIFI for the 33 biological targets extracted from the docking score ML data sets was conducted. AUPRC values, as depicted in Figure 7, revealed that FIFI was superior to PLEC and PLIF, while slightly inferior to ECFP. The superior performance of ECFP can be attributed to structurally analogous active compounds in the training and test data sets due to random splitting for training and test data set preparation. This contrasts with the SARM data sets, where a test data set contains a limited number of highly similar active compounds. Also, on another note, this kind of data set exhibits inherent compound series biases^68,69^ which are absent in the SARM data set. This bias arises from how the chemical compounds are generated in the drug discovery campaign and may give overoptimistic results for a certain prediction method. Nevertheless, when compared with IFPs, FIFI outperformed PLEC in 30 of the 33 targets. The Wilcoxon signed-rank test confirmed that the difference in AUPRC between FIFI and PLEC is statistically significant (*p* = 0.00004). It should be noted that both IFPs were derived from a similar core: ECFP, suggesting that any inherent bias should affect both approaches and not disproportionately enhance one fingerprint’s performance over another. FIFI takes the order of the amino acid residues in the binding pocket, and PLEC does not. The scores of each target and fingerprint are given in Table S5.

## 4 Conclusions

Appropriate methods for VS are important in any drug discovery project. Since various methods for ligand- and structure-based approaches including their combinations for VS have been proposed, a systematic comparison of the methods is necessary. In this study, we developed an IFP termed FIFI for hybrid VS approaches. FIFI incorporates ligand substructures within the vicinity of residues in a binding site. From a methodological point of view, FIFI keeps the order of amino acid residues in the binding site of a protein, which distinguishes itself from PLEC, a previously proposed IFP.

For our retrospective evaluation of VS approaches, structurally similar active compounds in a SARM were used as training, and a diverse test set of active and inactive compounds was screened for the six biological targets: ADRB2, Casp1, KOR, LAG, MAPK2, and p53. Our results showed that FIFI was superior to other IFP-based models, including PLEC and PLIF, and other VS approaches: sequential and parallel VS approaches. However, for KOR, ECFP-based ML models worked much better than other structure-based or hybrid approaches, irrespective of the identification of structurally (dis)similar active compounds. Thus, ECFP remains a viable fingerprint due to its simplicity and performance despite only using ligand-based information. On the other hand, PLIF showed poor performance in combination with ML models. Consensus scoring, despite its not-so-bad performances, gave little information about interpretability since it only consisted of two elements, ECFP and PLIF scores. It should be noted that FIFI and other methods incorporating ML methods are trained per target; therefore, these methods intrinsically rely on the availability of data with high quality.

FIFI introduced a way to address how to disclose integral information from both SBVS and LBVS parts. In drug design campaigns, hybrid VS methods, including FIFI, can be employed when both target and ligand information is available. Our results suggest that FIFI could be useful during the early stages of VS, especially in scenarios where the number of available active compounds is sparse and the compounds exhibit limited variability. In the future, FIFI needs to be improved in its IFP structure, for example, comprising a large sparse matrix, which prevents efficient computational calculations. The problem of the SBVS accuracy also needs to be addressed as one of FIFI’s critical components. In the future, we will also be exploring the possibility of developing a different translation method for these FIFI-generated substructures to other mediums besides fingerprints.

## Data Availability

A set of Python script files for calculating FIFI is provided in the GitHub repository: https://github.com/FIFI-VS/FIFI-FP. Descriptor sets for training and test compounds are provided in https://zenodo.org/records/13340333.

## Supporting Information Available

Four types of protein–ligand IFP (PLIF)-based representations, docking score (S score) distributions, AUPRC comparison among classification models for PLIF and ECFP4 representations, AUPRC comparison among classification models in combination with PLIF, ECFP4, and concatenated representations, average numbers of retrieved active compound scaffolds from the top 100 compounds using RF in whole and distinct data set, numbers of training and test compounds per SARM, numbers of test compounds per SARM based on Tanimoto similarity thresholds, interactions extracted by the MOE protein–ligand interaction (PLIF) module, docking score ML data sets used for fingerprint comparison, various fingerprints performance on the docking score ML data set, distributions of the docking scores (S) per compound category, median AUPRC values for the five classifier models in SARM general data set, and median AUPRC values for the two FIFI derivatives per the depth of neighboring atoms (PDF)
