# Supplementary material for: Bridging Structure- and Ligand-Based Virtual Screening through Fragmented Interaction Fingerprint
Source: ACS Omega. 2024 Sep 3;9(37):38957–69. doi: 10.1021/acsomega.4c05433 (PMC11411525; doi:10.1021/acsomega.4c05433)
Supplement: Supplementary file 1 — ao4c05433_si_001.pdf [file ao4c05433_si_001.pdf]

Supporting Information

Bridging Structure- and Ligand-based Virtual Screening through Fragmented Interaction Fingerprint (FIFI)

Rezi Riadhi Syahdi[1], Swarit Jasial[1,2], Itsuki Maeda[1], Tomoyuki Miyao*[1,2]

[1]Graduate School of Science and Technology, Nara Institute of Science and Technology, 8916-5 Takayama-cho, Ikoma, Nara, 630-0192, Japan

[2]Data Science Center, Nara Institute of Science and Technology, 8916-5 Takayama-cho, Ikoma, Nara, 630-0192, Japan

*Corresponding Author:

Email: miyao@dsc.naist.jp

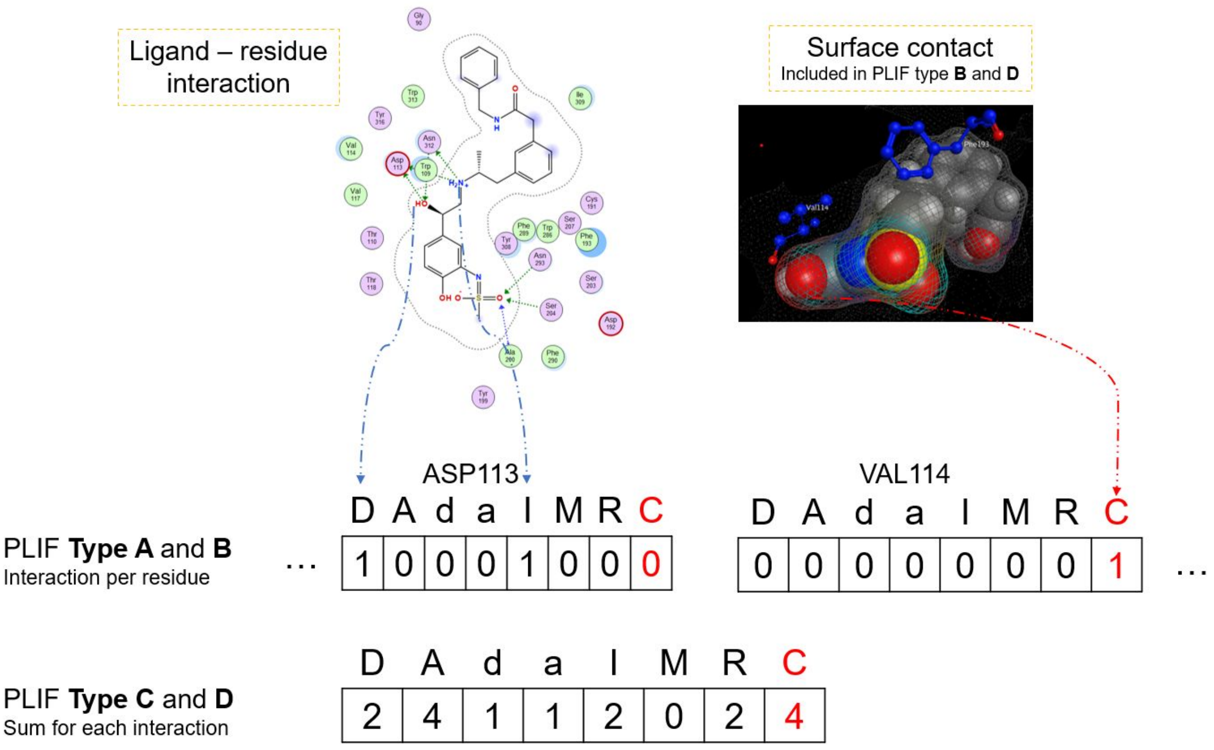

Figure S1. Four types of protein-ligand interaction fingerprint (PLIF)-based representations. Type A: interaction per residue; type B: interaction and surface contact per residue, type C: sum of interactions, type D: sum of interactions and surface contact

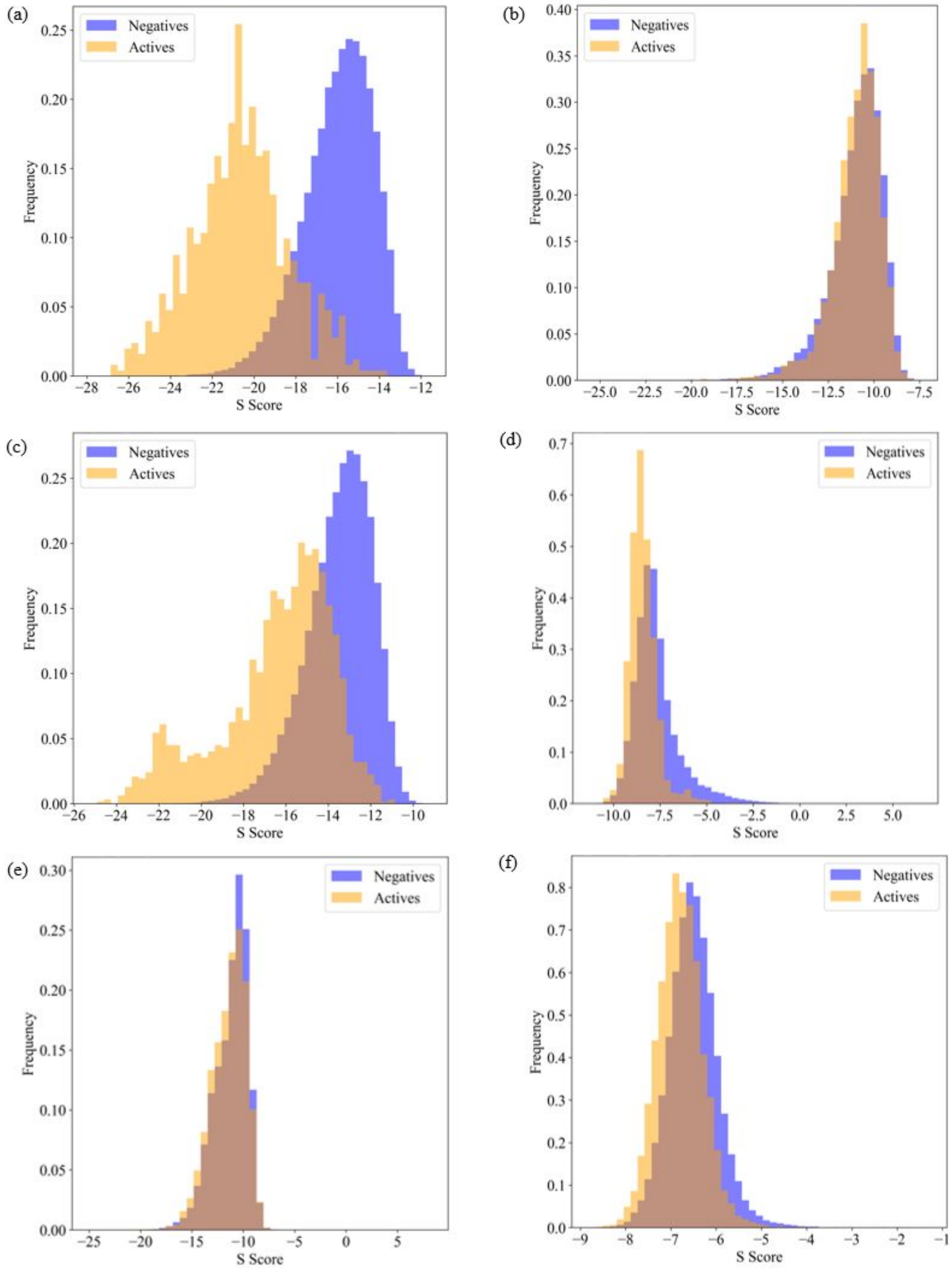

Figure S2. Docking score (S score) distributions. (a): beta-2 adrenergic receptor (ADRB2), (b) caspase-1 (Casp-1), (c) kappa-opioid receptor (KOR), (d) MAP kinase ERK2 (MAPK), (e) lysosomal alpha-glucosidase (LAG), (f) tumor suppresor p53. For each category (active or inactive), the distribution was scaled by the total number of samples.

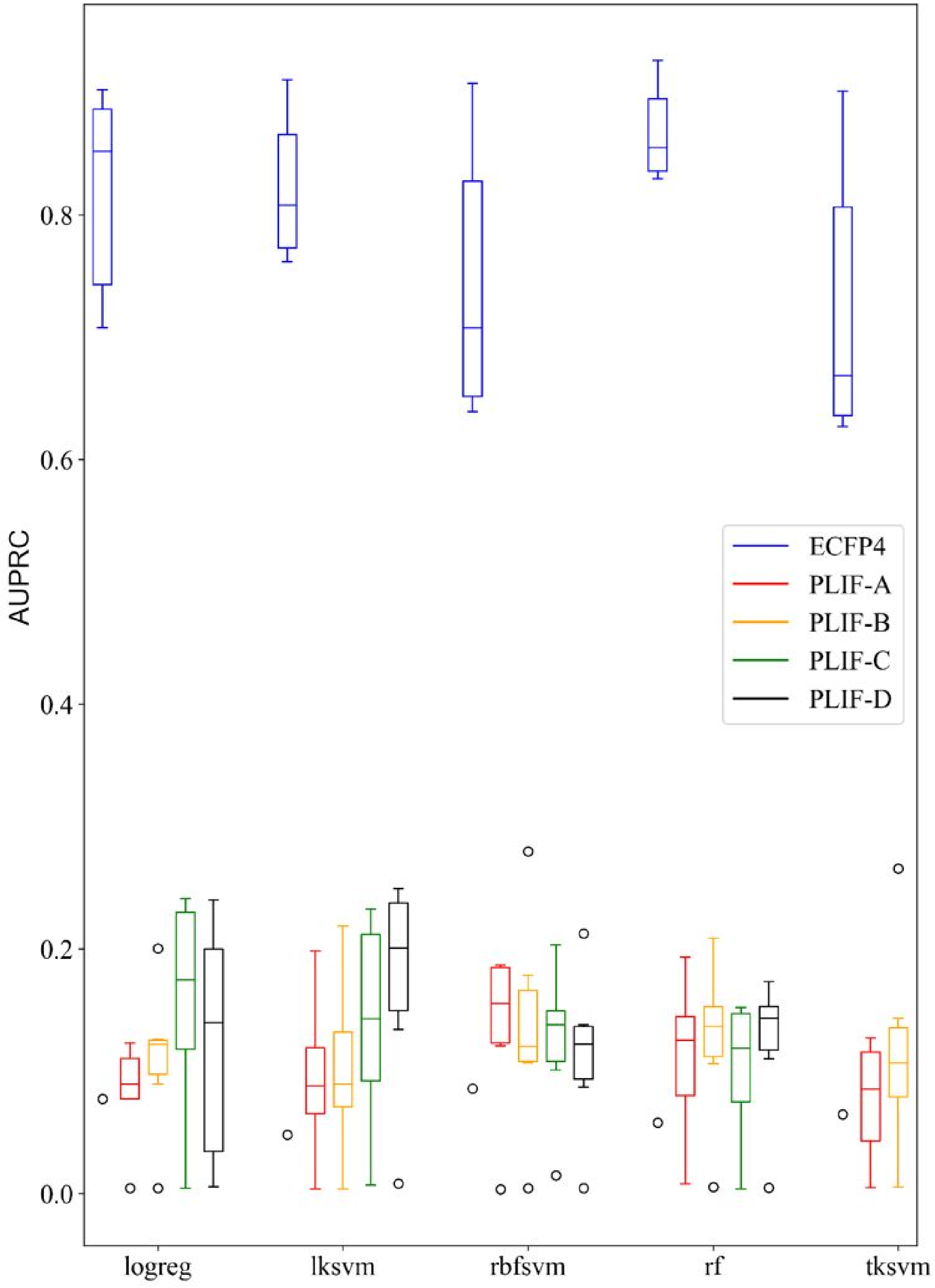

Figure S3. AUPRC comparison among classification models for PLIF and ECFP4 representations.

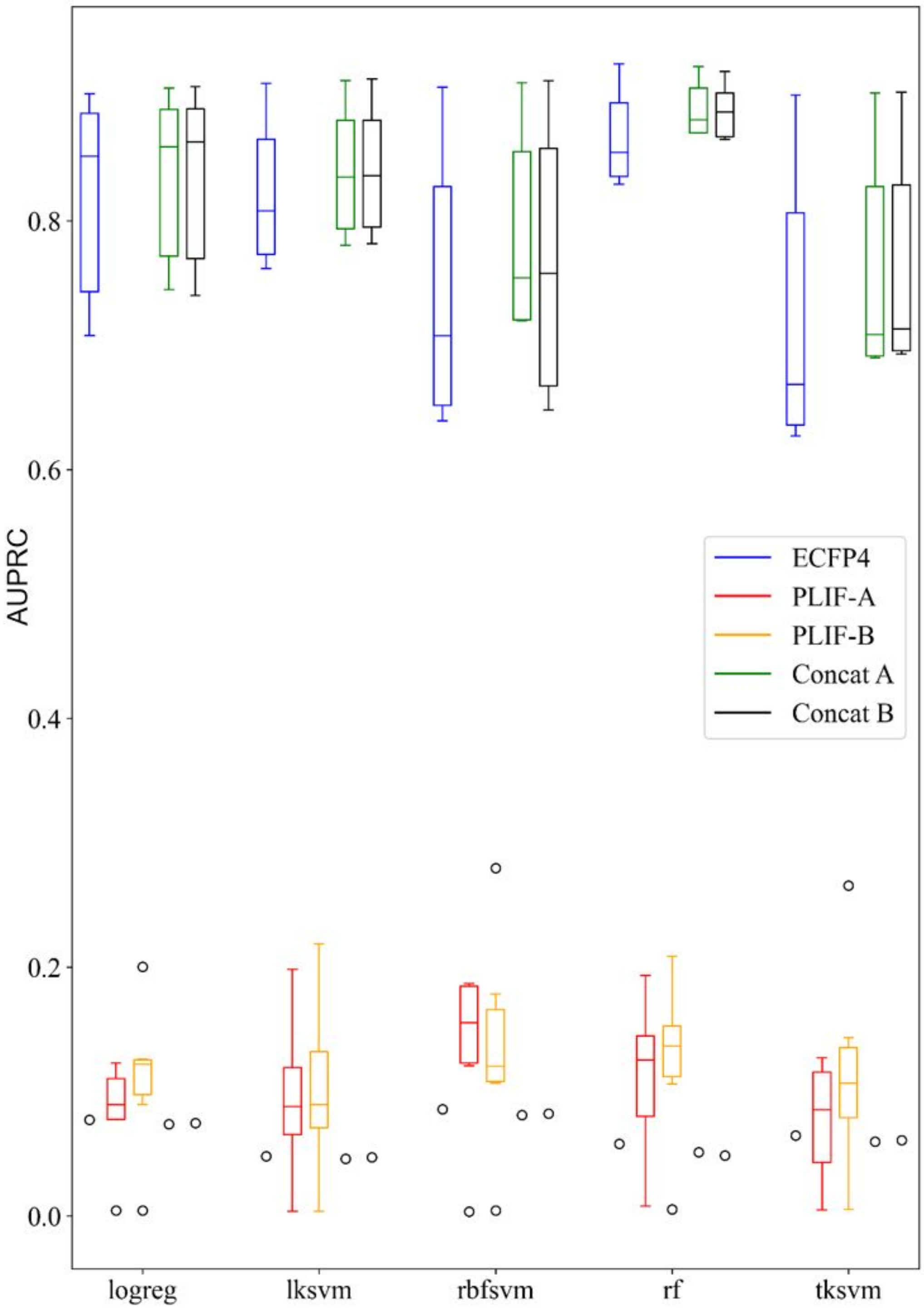

Figure S4. AUPRC comparison among classification models in combination with PLIF, ECFP4, and concatenated representations. PLIF type A is concatenated one-hot encoded interactions, PLIF type B is concatenated one-hot encoded interactions and surface contact. Concat A is concatenation of ECFP4 and PLIF type A, concat B is concatenation of ECFP4 and PLIF type B.

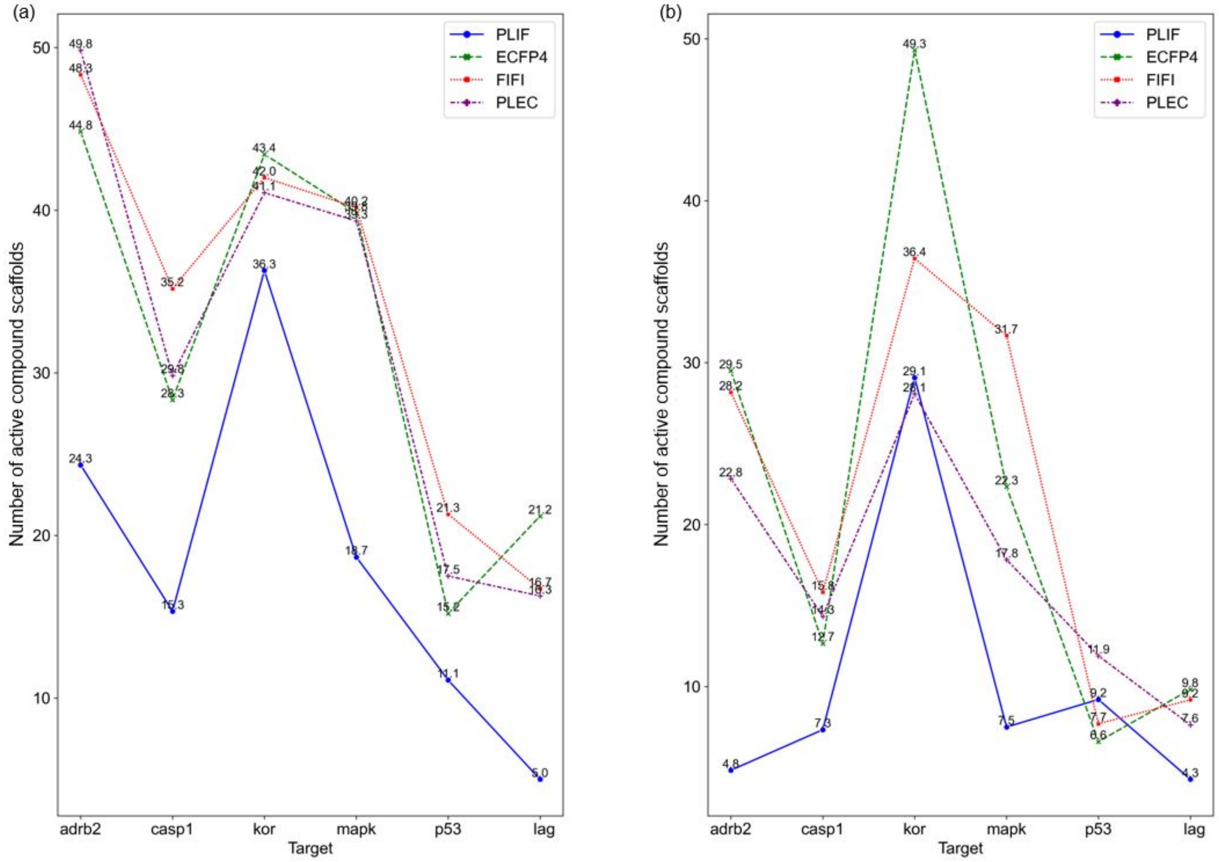

Figure S5. Average numbers of retrieved scaffolds from the top 100 compounds using RF in whole (a) and distinct (b) dataset.

Table S1 Numbers of training and test compounds per SARM.

SARMs	Active train.	Negative train.	Active test	Negative test
***Beta-2 adrenergic receptor* (ADRB2)**				
64486_series_1	30	1,500	731	104,669
77090_series_1	40	2,000	721	104,669
25601_series_1	45	2,250	716	104,669
40826_series_1	37	1,850	724	104,669
61635_series_1	19	950	742	104,669
79462_series_1	10	500	751	104,669
***Caspase-1* (Casp1)**				
8546_series_1	12	600	1,670	25,406
1811_series_1	13	650	1,669	25,406
2707_series_1	40	2,000	1,642	25,406
455_series_1	24	1,200	1,658	25,406
5_series_1	48	2,400	1,634	25,406
8593_series_1	12	600	1,670	25,406
***Map Kinase Erk1/2* (MAPK)**				
1_series_1	19	950	1,773	31,481
20999_series_1	14	700	1,778	31,481
5434_series_1	32	1,600	1,760	31,481
20655_series_1	18	900	1,774	31,481
13_series_1	24	1,200	1,768	31,481
258_series_1	39	1,950	1,753	31,481
***Kappa Opioid Receptor* (KOR)**				
22921_series_1	30	1,500	1,899	102,224
49752_series_1	30	1,500	1,899	102,224
65751_series_1	43	2,150	1,886	102,224
31775_series_1	10	500	1,919	102,224
90472_series_1	33	1,650	1,896	102,224
13965_series_1	29	1,450	1,900	102,224
78611_series_1	13	650	1,916	102,224
28852_series_1	24	1,200	1,905	102,224
46094_series_1	15	750	1,914	102,224
6346_series_2	19	950	1,910	102,224
4298_series_1	25	1,250	1,904	102,224
29097_series_1	15	750	1,914	102,224
9595_series_1	16	800	1,913	102,224
94741_series_1	10	500	1,919	102,224
**Lysosomal alpha-glucosidase (LAG)**				
114958_series_1	26	1,300	6,333	153,119
76849_series_1	14	700	6,345	153,119
30124_series_1	11	550	6,348	153,119
107196_series_1	16	800	6,343	153,119
8233_series_1	10	500	6,349	153,119

74730_series_1	13	650	6,346	153,119
45758_series_1	19	950	6,340	153,119
73507_series_1	11	550	6,348	153,119
77962_series_1	11	550	6,348	153,119
34145_series_1	10	500	6,349	153,119
58074_series_1	16	800	6,343	153,119
**Celullar tumor antigen p53**				
39603_series_1	35	1,750	7,703	90,000
57293_series_1	20	1,000	7,718	90,000
58608_series_1	19	950	7,719	90,000
55136_series_1	22	1,100	7,716	90,000
67522_series_1	40	2,000	7,698	90,000
34663_series_1	27	1,350	7,711	90,000
31689_series_1	25	1,250	7,713	90,000
65833_series_1	18	900	7,720	90,000
77922_series_1	17	850	7,721	90,000
71190_series_1	19	950	7,719	90,000

Table S2 Numbers of test compounds per SARM based on Tanimoto similarity thresholds.

SARMs	Tanimoto similarity < 0.35		Tanimoto similarity < 0.20	
	Active test	Negative test	Active test	Negative test
***Beta-2 adrenergic receptor* (ADRB2)**				
64486_series_1	659	104,647	156	86,618
77090_series_1	651	104,651	62	91,887
25601_series_1	586	104,666	85	92,784
40826_series_1	479	104,106	115	75,845
61635_series_1	716	104,295	408	72,528
79462_series_1	706	104,669	279	98,837
***Caspase-1* (Casp1)**				
8546_series_1	1,635	25,393	1,181	22,024
1811_series_1	1,592	25,272	1,114	19,229
2707_series_1	1,116	25,166	786	19,556
455_series_1	1,108	25,143	754	19,876
5_series_1	1,098	25,111	746	19,275
8593_series_1	1,650	25,284	1,171	20,421
***Kappa-opioid receptor* (KOR)**				
22921_series_1	1,781	102,224	1,719	100,330
49752_series_1	1,761	102,223	1,025	87,572
65751_series_1	1,600	102,216	1,065	91,170
31775_series_1	1,823	102,224	1,284	99,930
90472_series_1	1,880	101,642	1,808	85,616
13965_series_1	1,870	102,209	1,281	98,715
78611_series_1	1,876	102,082	1,295	76,468
28852_series_1	1,772	102,098	1,407	78,739
46094_series_1	1,809	102,183	1,236	90,663
6346_series_2	1,825	102,151	905	81,341
4298_series_1	1,829	101,955	1,061	60,531
29097_series_1	1,893	101,773	1,360	64,945
9595_series_1	1,868	102,223	1,317	90,553
94741_series_1	1,919	102,162	1,831	82,704
***Map Kinase Erk1/2* (MAPK)**				
1_series_1	1,686	31,480	684	29,486
20999_series_1	1,717	31,481	1,143	30,291
5434_series_1	1,519	31,473	207	26,504
20655_series_1	1,728	31,452	465	22,556
13_series_1	951	31,476	281	26,129
258_series_1	733	31,481	232	26,050
***Lysosomal alpha-glucosidase* (LAG)**				
107196_series_1	6,187	153,035	5,435	139,834
114958_series_1	6,318	153,074	5,835	133,749
30124_series_1	6,087	150,828	3,921	101,524
34145_series_1	6,324	152,878	5,926	142,700

45758_series_1	6,200	152,061	4,126	108,207
58074_series_1	6,321	151,596	5,512	127,164
73507_series_1	6,342	153,111	5,764	140,008
74730_series_1	6,315	153,043	5,392	141,163
76849_series_1	6,226	152,524	5,495	131,664
77962_series_1	6,292	152,751	5,567	132,723
8233_series_1	6,262	152,692	4,876	107,335
Celullar tumor antigen p53				
31689_series_1	7,502	89,210	3,192	47,350
34663_series_1	7,416	87,157	2,693	39,029
39603_series_1	7,619	88,907	4,362	53,606
55136_series_1	7,674	89,956	6,100	77,257
57293_series_1	7,558	89,417	2,752	48,592
58608_series_1	7,614	89,776	6,185	78,158
65833_series_1	7,469	88,383	3,584	48,561
67522_series_1	7,186	85,780	2,014	27,559
71190_series_1	7,579	88,845	4,810	55,926
77922_series_1	7,624	89,370	4,611	61,722

Table S3. Interactions extracted by the MOE protein-ligand interaction (PLIF) module.

Interaction	Abv	Weak threshold	Strong threshold	Unit
Hydrogen bond donor to sidechain	D	0.5	1.5	Kcal/mol
Hydrogen bond donor to backbone	d	0.5	1.5	Kcal/mol
Hydrogen acceptor from sidechain	A	0.5	1.5	Kcal/mol
Hydrogen acceptor from backbone	a	0.5	1.5	Kcal/mol
Solvent hydrogen bond	O	0.5	1.5	Kcal/mol
Ionic attraction	I	0.5	3.5	Kcal/mol
Metal ligation	M	0.5	3.5	Kcal/mol
Arene attraction	R	0.5	1.0	Kcal/mol
Total hydrophobic contact	C	15	25	$Å^2$

Table S4. Docking ML Score data set used for fingerprint comparison.

No	Target name	Target abbv	IC50 (mM)		N Train		N Test	
			Act (<)	Neg (>)	Act	Neg	Act	Neg
1	Vascular endothelial growth factor receptor 2	vegfr2	50	113	1,462	4,078	366	1,019
2	Janus kinase 2	jak2	22	50	1,276	2,687	319	672
3	Phosphoinositide 3-kinase alpha	pi3ka	61	148	985	2,623	246	657
4	Neurotrophic tropomyosin receptor kinase	ntrk	15	31	537	1,431	134	358
5	Insulin-like growth factor 1 (IGF-1) receptor	igf1r	11	46	341	885	86	221
6	c-MET tyrosin kinase	cmet	14	32	609	1,622	152	406
7	Janus kinase 3	jak3	30	100	713	1,890	178	473
8	Phosphoinositide-3-Kinase Regulatory	pi3kr	18	51	587	1,565	147	391
9	Phosphoinositide 3-kinase beta	pi3kb	175	540	400	1,047	100	261
10	FMS-like tyrosine kinase 3	flt3	21	52	442	1,150	111	287
11	fibroblast growth factor receptor-1	fgfr1	25	93	401	1,069	100	268
12	The nuclear transcription factor c-Jun	cjun	140	491	253	668	63	168
13	Fibroblast growth factor receptor-3	fgfr3	7	14	173	459	43	116
14	Ret protein	ret	25	160	132	348	33	87
15	Bromodomain Containing 4	brd4	131	430	493	1,270	123	318
16	Aurora-1	aurora1	8	19	227	577	55	145
17	Histone deacetylase 6	hdac6	40	124	354	941	88	236
18	AKT Protein kinase B	akt	22	69	542	1,402	136	351
19	Platelet-derived growth factor receptor	pdfgr	72	280	239	636	60	159
20	Erythrocyte sedimentation rate protein	esr	40	273	435	1,149	109	287
21	Fibroblast growth factor receptor 2	fgfr2	4	8	218	576	55	144
22	Fibroblast growth factor receptor 4	fgfr4	8	16	222	582	56	145
23	Poly [ADP-ribose] polymerase 1	parp1	23	50	445	1,131	111	283
24	B-raf protein	braf	6	15	690	1,754	173	438

25	Interleukin-1 receptor-associated kinase 4	irak4	8	22	377	995	94	250
26	Collagenase-3 / MMP-13	mmp13	4	10	414	1,109	103	278
27	indoleamine 2,3-dioxygenase	ido1	200	609	297	800	74	201
28	Dihydrofolate reductase	dhfr	209	628	161	427	40	107
29	Anaplastic lymphoma kinase	alk	12	40	239	638	60	160
30	Farnesyltransferase	ftase	18	84	336	900	84	225
31	Bruton's tyrosine kinase	btk	4	12	455	1,201	114	301
32	Janus kinase 1	jak1	4	6	770	1,917	193	479
33	Epidermal growth factor receptor	egfr	51	150	1,381	2,951	346	738

Table S5. Various fingerprints performance on Docking Score ML dataset

No	Target name	Target abbv	AUPRC				MCC			
			PLIF	PLEC	FIFI	ECFP	PLIF	PLEC	FIFI	ECFP
1	Vascular endothelial growth factor receptor 2	vegfr2	0.599	0.808	0.824	0.862	0.390	0.612	0.639	0.720
2	Janus kinase 2	jak2	0.630	0.843	0.845	0.905	0.348	0.624	0.642	0.725
3	Phosphoinositide 3-kinase alpha	pi3ka	0.618	0.827	0.853	0.910	0.416	0.632	0.684	0.761
4	Neurotrophic tropomyosin receptor kinase	ntrk	0.425	0.693	0.737	0.815	0.264	0.500	0.572	0.634
5	Insulin-like growth factor 1 (IGF-1) receptor	igf1r	0.744	0.944	0.947	0.976	0.474	0.777	0.828	0.899
6	c-MET tyrosin kinase	cmet	0.471	0.682	0.732	0.868	0.249	0.472	0.518	0.683
7	Janus kinase 3	jak3	0.681	0.917	0.935	0.957	0.445	0.806	0.827	0.841
8	Phosphoinositide-3-Kinase Regulatory	pi3kr	0.622	0.857	0.868	0.922	0.394	0.689	0.747	0.758
9	Phosphoinositide 3-kinase beta	pi3kb	0.631	0.856	0.859	0.872	0.473	0.699	0.691	0.722
10	FMS-like tyrosine kinase 3	flt3	0.497	0.737	0.769	0.827	0.201	0.459	0.532	0.578
11	fibroblast growth factor receptor-1	fgfr1	0.622	0.862	0.834	0.931	0.489	0.717	0.768	0.791
12	The nuclear transcription factor c-Jun	cjun	0.582	0.815	0.856	0.921	0.38	0.607	0.654	0.753
13	Fibroblast growth factor receptor-3	fgfr3	0.486	0.680	0.717	0.727	0.322	0.547	0.605	0.583
14	Ret protein	ret	0.533	0.726	0.736	0.823	0.373	0.575	0.631	0.66
15	Bromodomain Containing 4	brd4	0.484	0.813	0.849	0.850	0.304	0.68	0.728	0.758
16	Aurora-1	aurora1	0.539	0.768	0.804	0.824	0.281	0.524	0.633	0.643
17	Histone deacetylase 6	hdac6	0.583	0.857	0.874	0.906	0.399	0.656	0.723	0.780
18	AKT Protein kinase B	akt	0.589	0.829	0.826	0.882	0.373	0.699	0.689	0.759

19	Platelet-derived growth factor receptor	pdfgr	0.522	0.87	0.884	0.885	0.303	0.689	0.752	0.753
20	Erythrocyte sedimentation rate protein	esr	0.698	0.875	0.907	0.901	0.557	0.755	0.793	0.762
21	Fibroblast growth factor receptor 2	fgfr2	0.481	0.794	0.817	0.857	0.227	0.553	0.598	0.729
22	Fibroblast growth factor receptor 4	fgfr4	0.576	0.787	0.796	0.848	0.365	0.592	0.515	0.706
23	Poly [ADP-ribose] polymerase 1	parp1	0.593	0.832	0.841	0.871	0.360	0.684	0.708	0.732
24	B-raf protein	braf	0.650	0.854	0.862	0.880	0.410	0.698	0.710	0.743
25	Interleukin-1 receptor-associated kinase 4	irak4	0.830	0.904	0.918	0.908	0.692	0.818	0.806	0.835
26	Collagenase-3 / MMP-13	mmp13	0.435	0.736	0.811	0.833	0.145	0.513	0.588	0.672
27	Indoleamine 2,3-dioxygenase	ido1	0.587	0.831	0.790	0.830	0.425	0.660	0.617	0.704
28	Dihydrofolate reductase	dhfr	0.291	0.854	0.872	0.923	0.089	0.683	0.722	0.783
29	Anaplastic lymphoma kinase	alk	0.593	0.829	0.86	0.907	0.421	0.679	0.735	0.792
30	Farnesyltransferase	ftase	0.618	0.785	0.882	0.929	0.377	0.628	0.710	0.798
31	Bruton's tyrosine kinase	btk	0.538	0.699	0.715	0.783	0.337	0.491	0.524	0.602
32	Janus kinase 1	jak1	0.478	0.678	0.720	0.789	0.247	0.530	0.573	0.613
33	Epidermal growth factor receptor	egfr	0.586	0.846	0.884	0.946	0.312	0.662	0.708	0.810

Table S6. Distributions of the docking scores (S) per compound category.

(a) ADRB2

percentile	act_N	neg_N	act%	neg%	S threshold
0.1	74	42	63.79	36.21	-23.71
0.2	132	99	57.14	42.86	-22.83
0.5	240	338	41.52	58.48	-21.70
1	339	816	29.35	70.65	-20.87
2	456	1853	19.75	80.25	-20.14
5	591	5180	10.24	89.76	-19.10
10	648	10894	5.61	94.39	-18.27
20	699	22384	3.03	96.97	-17.34
50	743	56964	1.29	98.71	-15.78

(b) MAPK

percentile	act_N	neg_N	act%	neg%	S threshold
0.1	4	40	9.09	90.91	-10.33
0.2	7	80	8.05	91.95	-10.16
0.5	18	199	8.29	91.71	-9.96
1	32	401	7.39	92.61	-9.77
2	59	807	6.81	93.19	-9.55
5	170	1993	7.86	92.14	-9.25
10	351	3975	8.11	91.89	-8.97
20	723	7928	8.36	91.64	-8.64
50	1428	20200	6.60	93.40	-7.95

(c) Casp1

percentile	act_N	neg_N	act%	neg%	S threshold
0.1	1	37	2.63	97.37	-18.24
0.2	2	73	2.67	97.33	-17.64
0.5	8	178	4.30	95.70	-16.46
1	14	357	3.77	96.23	-15.61
2	27	715	3.64	96.36	-14.84
5	68	1786	3.67	96.33	-13.71
10	135	3573	3.64	96.36	-12.84
20	304	7111	4.10	95.90	-11.97
50	869	17667	4.69	95.31	-10.72

(d) KOR

percentile	act_N	neg_N	act%	neg%	S threshold
0.1	107	8	93.04	6.96	-21.85
0.2	194	35	84.72	15.28	-20.78
0.5	307	264	53.77	46.23	-19.24

1	393	749	34.41	65.59	-18.34
2	520	1763	22.78	77.22	-17.52
5	772	4934	13.53	86.47	-16.49
10	1018	10394	8.92	91.08	-15.67
20	1337	21486	5.86	94.14	-14.78
50	1775	55281	3.11	96.89	-13.32

(e) LAG

percentile	act_N	neg_N	act%	neg%	S threshold
0.1	9	159	5.36	94.64	-18.19
0.2	14	321	4.18	95.82	-17.57
0.5	31	807	3.70	96.30	-16.68
1	57	1618	3.40	96.60	-16.01
2	158	3192	4.72	95.28	-15.20
5	418	7955	4.99	95.01	-14.21
10	768	15978	4.59	95.41	-13.48
20	1491	32001	4.45	95.55	-12.62
50	3617	80111	4.32	95.68	-10.88

(f) p53

percentile	act_N	neg_N	act%	neg%	S threshold
0.1	21	87	19.44	80.56	-8.18
0.2	40	176	18.52	81.48	-8.05
0.5	108	431	20.04	79.96	-7.87
1	191	887	17.72	82.28	-7.75
2	374	1781	17.35	82.65	-7.60
5	890	4497	16.52	83.48	-7.38
10	1644	9129	15.26	84.74	-7.19
20	2866	18680	13.30	86.70	-6.96
50	5460	48404	10.14	89.86	-6.54

Table S7. Median AUPRC values for the five classifier models in SARMs general data set.

Target	Logreg	LKSVM	RBFSVM	TKSVM	RF
ECFP4					
ADRB2	0.852	0.808	0.707	0.668	**0.855**
Casp1	0.229	0.248	0.300	0.298	**0.308**
KOR	0.379	0.451	0.389	0.375	**0.702**
MAPK2	0.565	0.697	**0.701**	0.567	**0.701**
PLIF					
ADRB2	0.122	0.090	0.120	0.107	**0.136**
Casp1	0.070	0.070	0.071	0.070	**0.072**
KOR	0.147	**0.195**	0.150	0.128	0.163
MAPK2	0.125	0.126	**0.137**	0.103	0.115

Table S8. Median AUPRC values for the two FIFI derivatives per the depth of neighboring atoms.

Target	FIFI Concatenated Bits			FIFI Unique Substructures		
	N_0	N_1	N_2	N_0	N_1	N_2
ADRB2	0.842	0.871	0.891	0.846	0.858	0.880
Casp1	0.256	0.304	0.323	0.263	0.304	0.322
KOR	0.318	0.309	0.313	0.363	0.343	0.362
MAPK	0.691	0.733	0.767	0.721	0.755	0.780
